# Enhanced In-Vitro Hemozoin Polymerization by Optimized Process using Histidine-Rich Protein II (HRPII)

**DOI:** 10.3390/polym11071162

**Published:** 2019-07-08

**Authors:** Ju Hun Lee, Hyeong Ryeol Kim, Ja Hyun Lee, Soo Kweon Lee, Youngsang Chun, Sung Ok Han, Hah Young Yoo, Chulhwan Park, Seung Wook Kim

**Affiliations:** 1 Department of Chemical and Biological Engineering, Korea University, 145, Anam-Ro, Seongbuk-Gu, Seoul 02841, Korea; 2 Department of Food Science and Engineering, Dongyang Mirae University, 445, Gyeongin-ro, Guro-gu, Seoul 08221, Korea; 3 Department of Interdisciplinary Bio-Micro System Technology, College of Engineering, Korea University, 145 Anam-ro 5, Seongbuk-gu, Seoul 02841, Korea; 4 Department of Biotechnology, Korea University, 145, Anam-Ro, Seongbuk-Gu, Seoul 02841, Korea; 5 Department of Biotechnology, Sangmyung University, 20, Hongjimun 2-Gil, Jongno-Gu, Seoul 03016, Korea; 6 Department of Chemical Engineering, Kwangwoon University, 20, Kwangwoon-Ro, Nowon-Gu, Seoul 01897, Korea; 7 Department of Chemistry, Faculty of Science and Technology, Universitas Airlangga, Surabaya 60115, Indonesia

**Keywords:** biopolymers, heme, hemozoin, HRP-II, polymerase

## Abstract

Conductive biopolymers, an important class of functional materials, have received attention in various fields because of their unique electrical, optical, and physical properties. In this study, the polymerization of heme into hemozoin was carried out in an in vitro system by the newly developed heme polymerase (histidine-rich protein 2 (HRP-II)). The HRP-II was produced by recombinant *E. coli* BL21 from the *Plasmodium falciparum* gene. To improve the hemozoin production, the reaction conditions on the polymerization were investigated and the maximum production was achieved after about 790 μM at 34 °C with 200 rpm for 24 h. As a result, the production was improved about two-fold according to the stepwise optimization in an in vitro system. The produced hemozoin was qualitatively analyzed using the Fourier transform infrared (FTIR) spectroscopy, energy dispersive X-ray spectroscopy (EDS), and scanning electron microscopy (SEM). Finally, it was confirmed that the enzymatically polymerized hemozoin had similar physical properties to chemically synthesized hemozoin. These results could represent a significant potential for nano-biotechnology applications, and also provide guidance in research related to hemozoin utilization.

## 1. Introduction

Recently, conductive polymers have received attention in the nano-biotechnology industry because of their tremendous potential as a viable alternative to semi-conductive materials, or as energy sources for a variety of applications such as electronics, robots, energy devices, bio-chips, and composites [1,2,3,4,5,6]. In particular, the extent of the importance continues to increase, as the demand for next-generation displays and wearable electronic devices continues to grow. For example, conductive polymers containing porphyrin complexes, such as zinc-tetraphenylporphyrin (Zn), zinc-tetrabenzoporphyrin (Zn), iron-metalloporphyrins (Fe), ruthenium-metalloporphyrins (Ru), and osmium-metalloporphyrins (Os), are some of the well-known functional materials. These porphyrin structures show different metal centers incorporated within the tetradentate ligand, and the physical properties have a conformational flexibility of porphyrins with conductivity [7,8,9,10,11]. Thus, it is advantageous to develop advanced materials through control at the molecular level. Das and Prusty (2012) stated that future electronic nanodevices will be necessary in order to develop new functionalized polymers, because they require high durability, flexibility, and low resistance [1].

**Figure 1:**
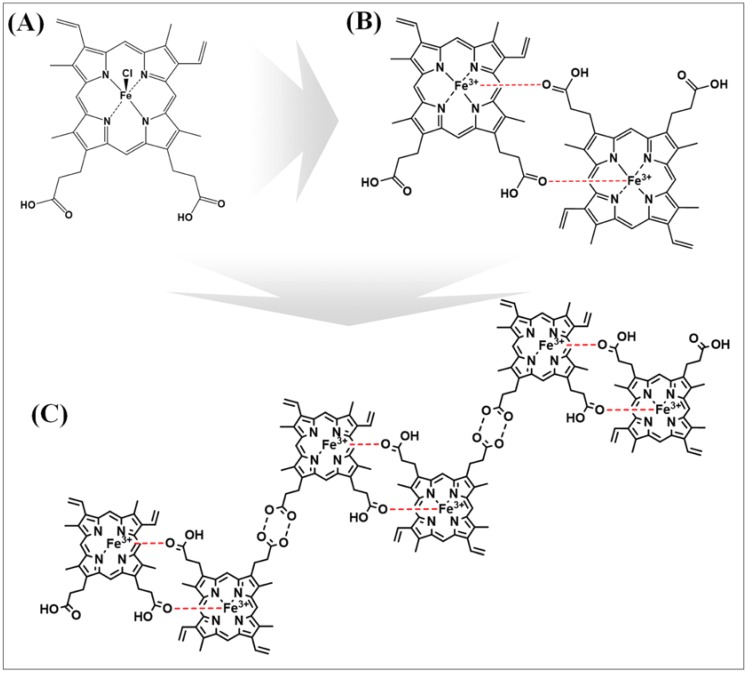
Schematic pathway of the hemozoin polymerization: (**A**) heme, (**B**) β-hematin, and (**C**) hemozoin.

Hemozoin was first reported in the early 18th century as a malaria pigment, but the final molecular structure was defined by an X-ray in 2000 [12,13]. Hemozoin has performed a decisive role in the discovery of the malarial parasite, as it utilizes a unique hemozoin formation pathway to avoid the heme toxicity. Figure 1 shows the structures of heme, β-hematin, hemozoin, and the mechanism of hemozoin polymerization. The polymerization reaction was carried out using the histidine-rich protein 2 (HRP-II) from the *Plasmodium falciparum* trophozoites. First, *Plasmodia* degraded the hemoglobin to produce the heme, which is then converted to the toxic free β-hematin by the HRP-II. The crystal structure of the β-hematin is considered to be structurally similar to hemozoin [12,13,14,15,16]. So far, most of the studies on the formation of hemozoin and its synthetic mechanism have been conducted in the medical science field [17,18]. Therefore, the electrochemical characteristics of hemozoin have not yet been reported for nano-biological applications.

Porphyrin, which is in contact with wide bandgap semiconductors, is attracting attention as a promising chromophore in materials application, because of its superior light absorption properties and large molar absorption coefficients [19,20,21]. Mousawi et al. (2017) reported that zinc tetraphenylporphyrin (ZnTPP) has been applied in new cationic 3D printing resins as a result of a number of major advantages (low temperature of usage, low emissions of volatile organic compounds, and low energy consumption) [7]. Following the report, heme, which is ferrous porphyrin complexes, has conductivity and biocompatibility. In particular, the polymerized heme nanocrystals showed a greater conductive property than a heme monomer [22]. Gargiulo et al. (2015) reported that new materials with biocompatibility and increased conductivity are applicable to the following areas: functional interfaces connecting biological systems to electrical devices, devices for translating optical and electronic stimuli into biological signals, devices for advanced cell culture systems, and devices for cell sorting and differentiation [23]. Therefore, a hemozoin featuring promising biocompatibility and an increased conductivity potentiality exists regarding nano-biomaterial applications.

Most of the studies on hemozoin polymerization were focused on the malaria detoxification in vivo [13,14,24,25]. To the best of our knowledge, this is the first investigation concerning the effects of the HRP-II reaction conditions on the hemozoin polymerization in vitro. In this study, the conductivity of the heme was firstly investigated in order to determine the potential applicability of hemozoin using a two-point probe measurement. The polymerization of hemozoin was performed using HRP-II, and the effect of the reaction conditions such as the initial enzyme concentration, agitation speed, reaction time, and temperature were investigated based on the amount of polymerization. The qualitative analysis of the produced hemozoin was confirmed using Fourier transform infrared (FTIR) spectroscopy, energy dispersive X-ray spectroscopy (EDS), and scanning electron microscopy (SEM), which were applied to evaluate the characteristics, and these results will be the basis for the nano-biomaterial utilization of hemozoin.

## 2. Experimental

### 2.1. Materials

The heme (powder from bovine ≥90%) and dimethyl sulfoxide (DMSO; liquid for plant cell culture tested ≥99.5%) were purchased from Sigma-Aldrich (St. Louis, MO, USA). The sodium phosphate (Na_3_PO_4_), sodium hydroxide (NaOH), and sodium bicarbonate (NaHCO_3_) were purchased from Samchun Pure Chemical (Kangnam-Gu, Seoul, South Korea).

### 2.2. Biological Experiments

#### 2.2.1. Expression and Production of the HRP-II

The gene cloning, expression, and purification of HRP-II have been studied elsewhere. The HRP-II genomic DNA was cloned from *Plasmodium falciparum* using the polymerase chain reaction (PCR). The primer sequences 5′-CCGGAATTCATGAATAATTCCGCATTTAAT-3′ (HRP-II F) and 5′-GCCGACGTCGACTTAATGGCGTAGGCAATG-3′ (HRP-II R) were designed on the basis of studies of the deoxyribonucleic acid (DNA) sequences of the HRP-II [22,26]. Thereafter, the plasmid was transformed into the *Escherichia coli* BL21(DE3). The HRP-II expression in *E. coli* BL21 (DE3) was kindly provided by the Molecular Microbial Biotechnology and Bioenergy Laboratory in Korea University. The main cultures were prepared in 500 mL of a lysogeny broth (LB) medium (1% tryptone, 0.5% yeast extract, and 1% sodium chloride (NaCl)) that was incubated at 37 °C until an optical density (O.D) of 0.6 was reached in the shaking incubator at 180 rpm. Then, 0.5 mM of isopropyl β-d-1-thiogalactopyranoside (IPTG) was included in the main culture at 17 °C for 12 h in a shaking incubator at 180 rpm. In addition, the medium contained 50 μg/mL of ampicillin [22].

#### 2.2.2. Enzyme Purification

The cells were collected using a centrifuge at 4000× *g* at 4 °C for 30 min. The collected cells were disrupted in order to obtain the intracellular protein in the lysis buffer (50 mM monosodium phosphate (NaH_2_PO_4_), 300 mM NaCl, and 10 mM imidazole, as well as NaOH with a pH of 8.0 that changes) using a sonicator. The cell debris-free supernatants were collected using centrifugation at 8000× *g* at 4 °C for 30 min.

The supernatants were concentrated using an ultrafiltration membrane (10 kDa; Sartorius, Gottingen, Germany). The concentrated supernatants were loaded into a chromatography column (Econo-column, Bio-Rad, Hercules, CA, USA) packed with nickel–nitrilotriacetic acid (Ni–NTA) resin (Qiagen, Hilden, Germany) at 1 mL/min. Then, the column was loaded with a washing buffer (50 mM NaH_2_PO_4_, 300 mM NaCl, and 20 mM imidazole, and the pH was adjusted to 8.0 with NaOH) at 2 mL/min. Finally, the enzyme (HRP-II) was collected using an elution buffer (50 mM NaH_2_PO_4_, 300 mM NaCl, 10 mM imidazole, 0.005% Tween 20, and NaOH with a pH of 8.0 that was adjusted) at 2 mL/min [27,28,29,30].

### 2.3. Heme to the Synthesis of Hemozoin

#### 2.3.1. Polymerization of the Heme

The heme powders were dissolved in 0.1 M NaOH, and then the 10 mM of the heme solution was filtered using the 0.2 μm PTFE membrane filters (hiPTFE membrane, G1320, GENIE, Taiwan). The polymerization of the 10 mM heme solution was performed using HRP-II in a 0.1 M Na_3_PO_4_ buffer with a pH of 6 at 37 °C for 18 h in a shaking incubator. The protein concentration was measured using the Bradford method from the absorbance at 595 nm [31].

#### 2.3.2. Estimation of the Hemozoin

The enzymatic reaction was stopped by the addition of 10 μL of sodium dodecyl sulfate (SDS), and then, the sample was centrifuged by 8000× *g* at 25 °C for 1 h. The supernatant was removed and then the precipitate was suspended with NaHCO_3_ (100 mM, pH 9) in a 2.5% SDS solution. This step was performed for the dissolution of most of the free heme and its by-products during the assay. Finally, the hemozoin was collected by centrifugation at 8000× *g* at 25 °C for 1 h. The hemozoin was dissolved in 20 mM NaOH to quantify its concentration, which is estimated from its absorbance at 400 nm [15,26].

### 2.4. Physico-Chemical Measurements

The conductivity, which was taken to be (σ = J/E_0_), was measured using a voltmeter with a sensitivity of 0.01 mV (Keithley, Model 248 High Voltage Supply). The FTIR spectroscopy analysis was performed to identify the functional group with a Frontier spectrometer (PerkinElmer). Potassium bromide (KBr) was mixed with the samples in an attempt to achieve an optical transparency for the light in the range of the FTIR measurements. SEM was confirmed to analyze the morphologies using a field emission gun scanning electron microscopy (FEG-SEM) instrument (Inspect F50, FEI), provided with an energy dispersive X-ray spectroscopy (EDS) detector (APOLLO XL, EDAX) at an accelerating voltage of 15 kV [32].

## 3. Results and Discussion

### 3.1. Comparison of the Conductivity of Various Porphyrin Complexes and the Heme

Recently, porphyrin functionalized complexes have been used in various applications of conductive materials, such as storage, memories, transistors, solar energy devices, molecular sensors, and low-dimensional conductors. The conductivity values of the synthesized conductive materials were measured in order to evaluate the electrochemical properties. The porphyrin complexes are very similar to the heme in structure. Currently, porphyrin complexes are frequently used in many conductive materials, and a heme with a similar structure has the potential to be useful as a conductive material in the future. However, the conductivity of heme (monomer of hemozoin) has not been reported yet. The conductivity (siemens) of heme and hemozion was found to be 4.0 × 10^−3^ μS/cm and 6.2 × 10^−3^ μS/cm, respectively (Table 1). Kobayashi et al. (1993) reported that the conductivity of the synthesized tetraphenylporphyrin (Zn) was less than 1.0 × 10^−5^ μS/cm. The similar functionalized complex, tetrabenzoporphyrin (Zn), was reported as about 4.0 × 10^−4^ μS/cm [33]. The metalloporphyrin functionalized complexes were studied, and their conductivities were reported by Collman et al. (1986) [10]. The results were as follows: metalloporphyrins (Fe), 2.8 × 10^−5^ μS/cm; metalloporphyrins (Ru), 1.0 × 10^−5^ μS/cm; and metalloporphyrins (Os), 8.0 × 10^−5^ μS/cm. Among them, tetrabenzoporphyrin (Zn) showed the highest conductivity of 4.0 × 10^−4^ μS/cm [10,33]. However, the prepared heme showed about 10-fold higher conductivity than tetrabenzoporphyrin (Zn). The heme is one of the redox-active metal complexes, and its electrochemistry has been extensively researched. Consequently, it was applied to various unique functions mimicking the heme proteins in biological systems. Following the reports, the heme (ferrous porphyrin complexes) has good conductivity and biocompatibility. It shows the different metal centers incorporated within the tetradentate ligand and the physical properties have a conformational flexibility of porphyrins with conductivity [7,8,9,10,11]. In addition, the heme has been found to have an oxidation-reduction activity, and it has also been reported to have a great influence on electron transfer under an oxidation-reduction reaction [34,35]. In this result, the polymerized hemomzoin nanocrystals showed a 1.5-fold improved conductive property than the heme monomer. Therefore, it is expected that hemozoin, a polymeric material from heme, could also enhance the physical properties and could be an alternative to the porphyrin complexes in nano-biomaterial applications such as organic and nanoscale electronics, and single-molecule- and nanomagnetism-based devices [8,22].

### 3.2. Production of the HRP-II and its Application to the Hemozoin Polymerization

THe HRP-II expression into *E. coli* BL21 (DE3) was selected on an LB agar (LBA) plate, including 50 µg/mL of ampicillin. The cell was inoculated to the LB media with 0.5 mM IPTG. The cells were subsequently collected and disrupted to gain the intracellular protein, and the cell debris-free supernatants were purified using the Ni–NTA agarose. The purification details are shown in Table 2. When HRP-II was purified, the specific activity of the crude broth was 57 U/mg-protein, and it was purified by a 2.5-fold extent with a recovery yield of 79.1%, using Ni–NTA affinity column chromatography.

**Figure 2:**
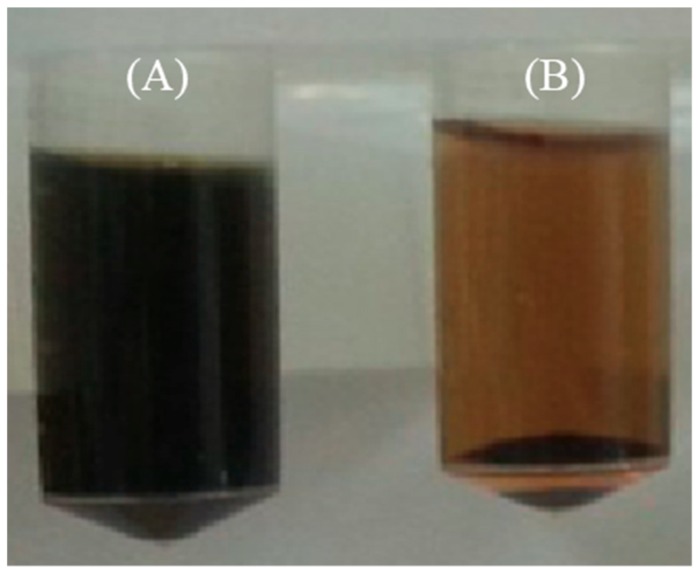
Photographs of before and after enzymatic reaction in an in vitro system. (**A**) Control group without enzyme (**B**) and experimental group with histidine-rich protein 2 (HRP-II).

The produced HRP-II was applied to the polymerization of hemozoin in an in vitro system. The enzymatic reaction was performed using the 1 mM heme as a substrate at 37 °C for 18 h. A control experiment was performed without the addition of the enzyme, and 1.5 μg/mL of the HRP-II was added in the experimental group to confirm the polymerization (Figure 2). As a result, the polymerized hemozoin (B) was confirmed in the experimental group, and the concentration of hemozoin was measured to be about 300 μM. Whereas, the hemozoin could not be confirmed in the control group (A, without enzyme).

### 3.3. Qualitative Characterization of the Polymerized Hemozoin

**Figure 3:**
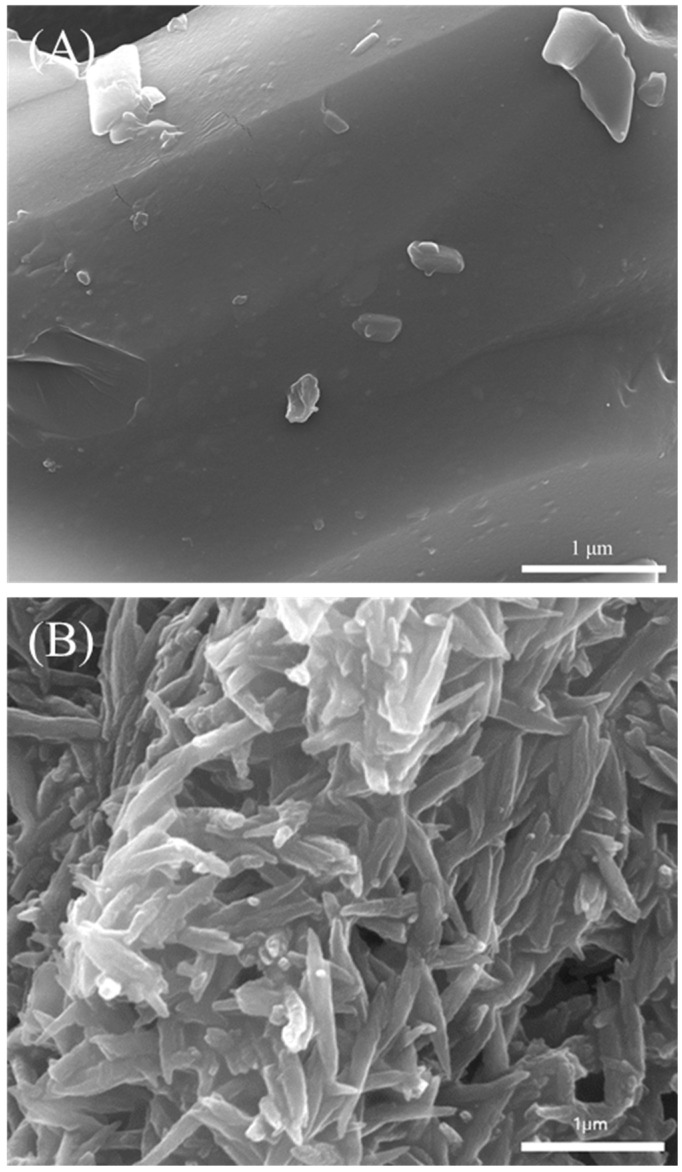
Representative field emission gun-scanning electron microscope (FEG-SEM) images of (**A**) heme and (**B**) polymerized hemozoin.

To compare the chemically identified hemozoin with the produced material (polymerized by HRP-II), a qualitative characterization was carried out using SEM, FTIR, and EDS. SEM was performed to observe the change of the polymerization with the crystal shapes and morphologies. The control group (heme) and the polymerized hemozoin by HRP-II is shown in Figure 3. The heme could not observe any pattern, however, the polymerized hemozoin showed a typical crystal morphology and was very similar to the structure of the chemically identified hemozoin or that synthesized in vivo by *Plasmodium* as reported from other studies [13,24]. This structure is known as ferriprotoporphyrin IX (Fe(III)PPIX) in nomenclature [36].

**Figure 4:**
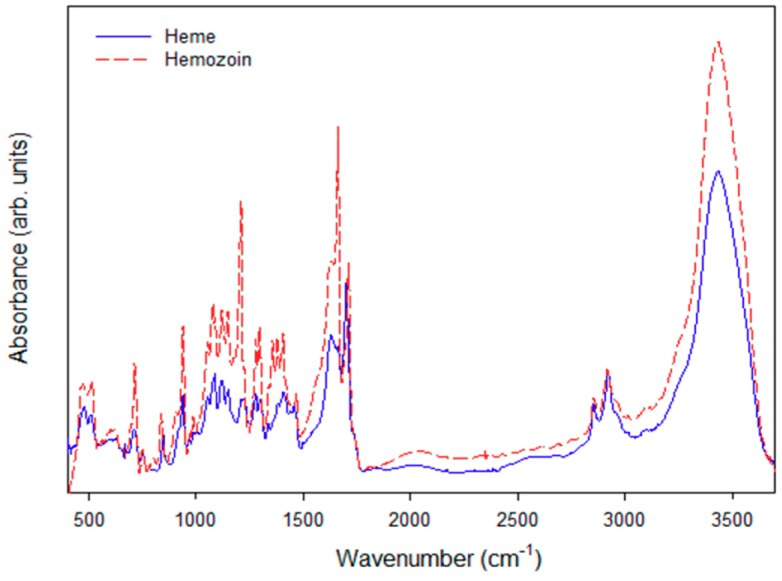
Fourier transform infrared (FTIR) spectra of heme and polymerized hemozoin.

The spectra of the heme and hemozoin by FTIR analysis are shown in Figure 4. The spectra show the chemical compositions of the heme to hemozoin polymerization reaction peaks for each level. The heme and hemozoin were confirmed by the characteristic FTIR peaks that appeared at 3300–3400 (O–H stretching), 3000 (C–H stretching), 1700–1800 (C=O stretching), 1660 (C=C stretching), 1460 (–CH_2_ stretching), 1100~1200 (C–O and C–C stretching), and 800 (N–H stretching) using FTIR. The peaks of the O–H, C=C, C–C, C–O, and N–H peaks were significantly indicated for the hemozoin. Tempera et al. (2015) reported on the FTIR spectra of the chemically identified hemozoin. The FTIR spectra was similar to the polymerized hemozoin by HRP-II in the current study [37]. Thus, it was confirmed that hemozoin polymerization was performed successfully.

The elemental compositions of the heme and polymerized hemozoin were investigated using an EDS detector. The results of the elemental composition are indicated in Table 3. The carbon (C) composition was confirmed as 71.14% and 75.63% for heme and hemozoin, respectively. Also, the oxygen (O) composition was confirmed as 7.35% and 9.56%, and the iron (Fe) was confirmed as 4.27% and 9.81% for heme and hemozoin, respectively. As a result, the compositions in hemozoin were at about a 1.15-fold increase compared with the total heme. These quantitative analyses confirmed that the material polymerized by the HRP-II was similar to the chemically identified hemozoin, and thus, the produced hemozoin by the HRP-II has potential in nano-biomaterial applications.

### 3.4. Effects of the Reaction Conditions on Hemozoin Polymerization

**Figure 5:**
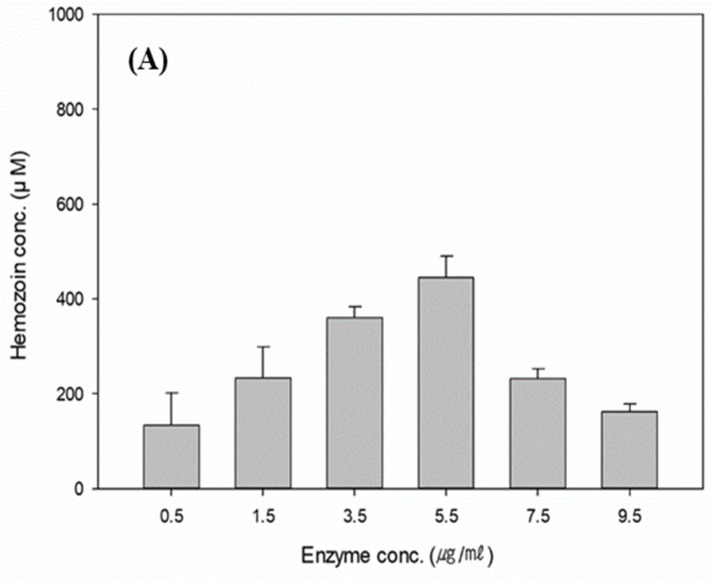

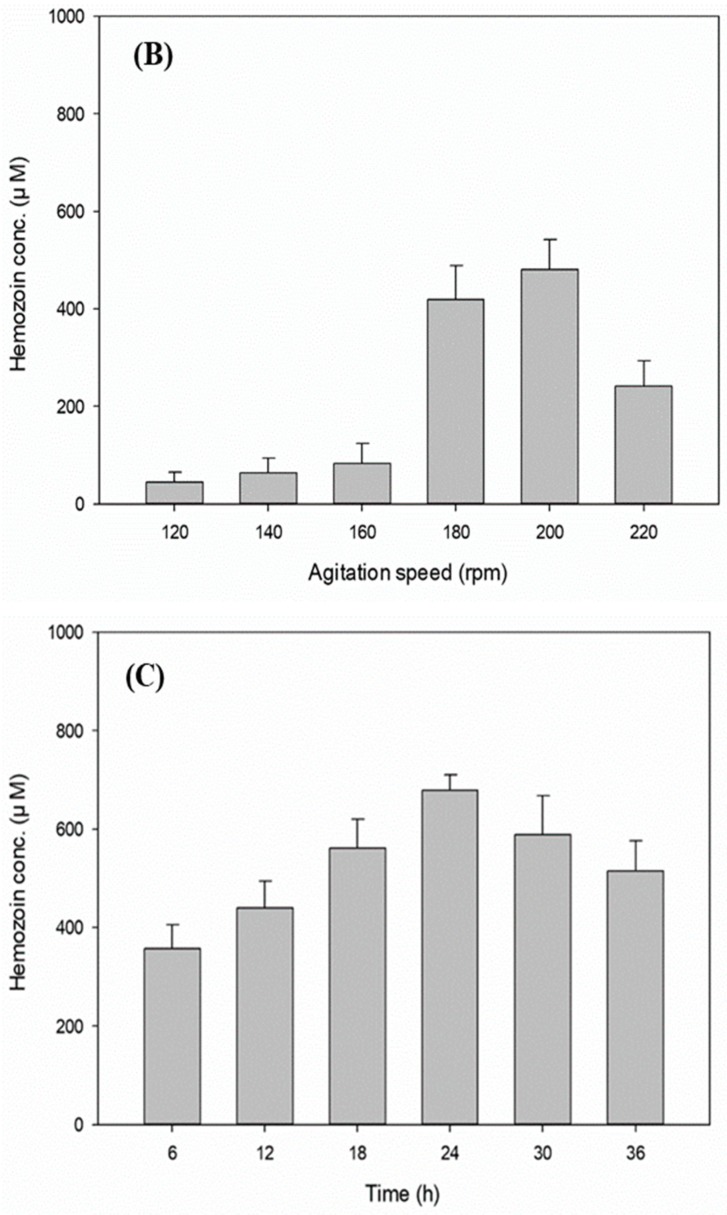

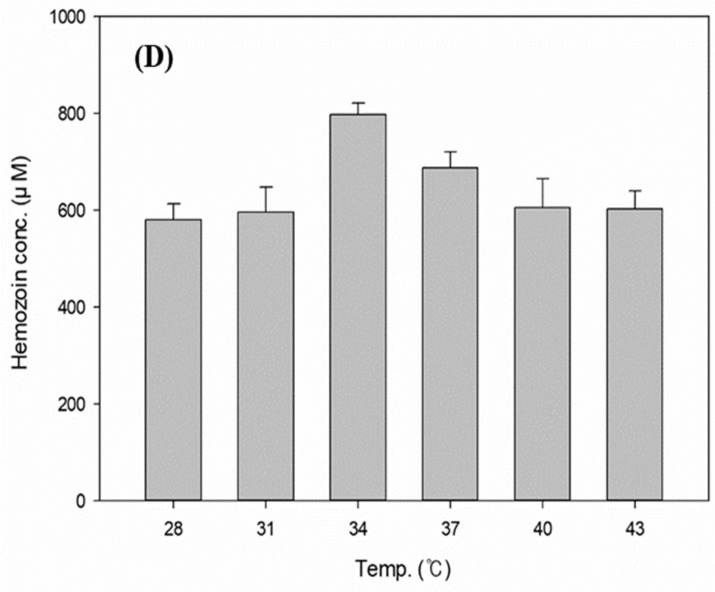
Effects of the reaction conditions on hemozoin concentration. (**A**) Initial enzyme loading, (**B**) agitation speed, (**C**) reaction time, and (**D**) reaction temperature.

In the previous section, the hemozoin was successfully produced by the HRP-II, and the characterization was confirmed using a quantitative analysis. Many studies have been reported about the detection of hemozoin in vivo, whereas it has been rarely reported for polymerizations in vitro. In particular, the production of hemozoin for nano-bioindustrial application has not been studied. In the current study, the enzymatic polymerization of hemozoin was fundamentally investigated in the system. Here, the effect of the initial HRP-II loading on the polymerization ratio and hemozoin concentration was preferentially studied. The polymerization conditions were performed in a 10 mM heme solution with a 0.1 M Na_3_PO_4_ buffer (pH 5.5) at 37 °C, 180 rpm for 18 hr. The HRP-II concentrations were used in the range of 0.5 to 9.5 μg/mL. Figure 5A shows the relationship between the HRP-II loading and the produced hemozoin. The hemozoin was significantly polymerized according to the increasing of the HRP-II concentration. However, hemozion polymerization was decreased at 5.5 μg/mL HRP-II and above. Because, as the enzyme concentration increases, the substrate–enzyme reaction proceeded to competitive inhibition, and the concentration of hemozoin was spontaneously reduced when the enzyme reaction rate decreased [38]. Finally, the hemozoin concentration that was produced by the use of 10 mM heme is 440 μM less than the 5.5 μg/mL of the enzyme level (HRP-II), and its activity and specific activity were found to be 0.317 units and 57 unit/mg of protein, respectively.

The mass transfer is an important factor affecting the rate of polymerization in heterogeneous polymerization systems. The effects of the agitation speed on the polymerization ratio and the hemozoin concentration were also investigated, as shown in Figure 5B. The enzyme reaction was performed using 5.5 μg/mL HRP-II in a 10 mM heme solution with a 0.1 M Na_3_PO_4_ buffer (pH 5.5) at 37 °C for 18 h. The agitation speed was adjusted from 120 to 220 rpm. The hemozoin concentration was steadily increased with stirring speed, from 120 to 200 rpm. Finally, the maximum concentration of hemozoin (480 μM) was obtained at 200 rpm, beyond which it decreased. This is because the substrate–enzyme reaction was inhibited during the high agitation speed. Accordingly, the requisite optimal agitation speed required to attain a high conversion was 200 rpm rather than at another agitation speed. Arai et al. (1981) and Naghash et al. (2007) reported that a similar tendency has been observed in emulsion polymerization without a solid phase. One of the most important factors is considered to be a mass transfer on the polymerization of a monomer in the aqueous phase. Accordingly, the effect of oxygen is also an important factor in the polymerization. The oxygen inhibition also increases with the increase of the agitation speed. Therefore, the polymerization gradually decreases as the stirring speed is increased [39,40]. The effects of the reaction time on the polymerization ratio and hemozoin concentration were also investigated, as shown in Figure 5C. The enzymatic reaction was performed using the 5.5 μg/mL HRP-II in the 10 mM heme solution with the 0.1 M Na_3_PO_4_ buffer (pH 5.5) at 37 °C and 200 rpm. The experiment for the reaction time was designed as 6, 12, 18, 24, 30, and 36 h. The hemozoin concentration was found to increase until 24 h, and the maximum production of hemozoin, of about 679 μM, was achieved at 24 h. However, over the 24 h reaction, the de-polymerization of hemozoin was observed, as it is believed that the reaction is composed of reversible reactions. As a result, the concentration of hemozoin was spontaneously broken according to the increase of the polymerization rate. The effects of the reaction temperature on the polymerization ratio and hemozoin concentration were also studied. The polymerization conditions were performed in a 10 mM heme solution with a 0.1 M Na_3_PO_4_ buffer (pH 5.5) at 200 rpm for 24 h. Figure 5D presents the relationship between the reaction temperature and the produced hemozoin, and the experiments were performed at 28, 31, 34, 37, 40, and 43 °C. Overall, the reaction temperature was not significantly affected compared with the other factors. The maximum production of hemozoin was about 790 μM at 34 °C.

### 3.5. Comparison of the Polymerization Yields under the Determined Conditions

**Figure 6:**
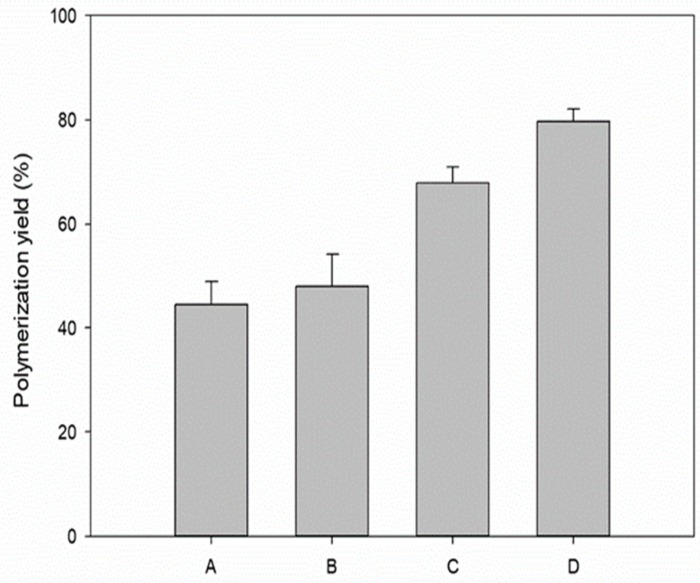
Summary of the polymerization yield under the determined conditions. (A: 5.5 μg/mL HRP-II, 180 rpm, 18 h, 37 °C; B: 5.5 μg/mL HRP-II, 200 rpm, 18 h, 37 °C; C: 5.5 μg/mL HRP-II, 200 rpm, 24 h, 37 °C; D: 5.5 μg/mL HRP-II, 200 rpm, 24 h, 34 °C).

The effect of the enzyme reaction conditions on the polymerization of hemozoin was successfully investigated in Section 3.4. The concentration of the polymerized hemozoin was significantly affected by the reaction factors. Figure 6 shows the correlation between the determined condition at each stage (A to D) and the yield of hemozoin polymerization. In stage A, the maximum production of hemozoin (440 μM) was achieved at 5.5 μg/mL HRP-II, and the yield of polymerization was about 44%. The agitation speed was determined as 200 rpm at stage B. The maximum yield of polymerization was about 48%. Condition C, the effect of the reaction time, was determined at 24 h, and the yield of polymerization was about 68% to the maximum. Finally, the maximum yield of hemozoin polymerization was about 80% at condition D. As a result, the increasing of the polymerization yield was confirmed with each stage, according to the optimization. In summary, the polymerization of hemozoin by HRP-II was about two-fold improved at optimal conditions. For the application of hemozoin in nano-bioindustry, more studies are required, such as reaction optimization by statistical method, reutilization of HRP-II, design of reactor system, and so on.

## 4. Conclusions

Hemozoin, polymerized from the heme, has shown a great potential as a new nano-biomaterial in the near future, as it has a similar structure to porphyrin, used in conductive materials. The conductivity of heme was preferentially estimated as 4.0 × 10^−3^ μS/cm by two-point probe measurement. The production and purification of HRP-II were carried out and finally purified to 2.5-fold with a recovery yield of 79.1%, using Ni–NTA affinity column chromatography. The polymerization of hemozoin using HRP-II was performed under each condition, and the qualitative analysis of the produced hemozoin was confirmed by FTIR, EDS, and SEM. These quantitative analyses confirmed that the material polymerized by HRP-II was similar to the chemically identified hemozoin. The in vitro conversion of hemozoin has not been studied, therefore, the reaction conditions on the hemozoin polymerization were fundamentally investigated to improve the production process. The maximum production was about two-fold improved (790 μM) by the stepwise optimization (34 °C, 200 rpm for 24 h). To our experience, this is the first research where the in vitro polymerization of hemozoin has been investigated under various conditions. Therefore, this research could provide useful information for other inquiries related to hemozoin applications.

## Figures and Tables

**Table 1:** Comparison of the conductivity of various porphyrin complexes, heme, and hemozoin.

Conductive Polymer	Chemical Formula	Conductivity (μS/cm)	Ref.
Metalloporphyrins (Fe)	C_44_H_30_N_4_Fe	2.8 × 10^−5^	[10]
Metalloporphyrins (Ru)	C_44_H_30_N_4_Ru	1.0 × 10^−5^	[10]
Metalloporphyrins (Os)	C_44_H_30_N_4_Os	8.0 × 10^−5^	[10]
Tetraphenylporphyrin (Zn)	C_44_H_30_N_4_Zn	<1.0 × 10^−5^	[33]
Tetrabenzoporphyrin (Zn)	C_36_H_22_N_4_Zn	4.0 × 10^−4^	[33]
Heme	C_34_H_32_ClFeN_4_O_4_	4.0 × 10^−3^	This study
Hemozoin	–[C_34_H_32_ClFeN_4_O_4_]–	6.2 × 10^−3^	This study

**Table 2:** Purification summary of histidine-rich protein 2 (HRP-II). Ni–NTA—nickel–nitrilotriacetic acid.

Purification Step	Total Protein (μg)	Total Activity (U)	Specific Activity (U/mg)	Yield (%)	Purification (folds)
Crude broth	17.8	0.40	22.5	100.0	1.0
Ni–NTA affinity column	5.5	0.32	57.0	79.1	2.5

**Table 3:** Composition of various element contents using an energy dispersive X-ray spectroscopy (EDS) detector.

Element	Carbon (C) (%)	Oxygen (O) (%)	Iron (Fe) (%)
Heme	71.14	7.35	4.27
Hemozoin (polymerized by HRP-II)	75.63	9.56	9.81
